# Non-conventional Ce:YAG nanostructures via urea complexes

**DOI:** 10.1038/s41598-019-39069-6

**Published:** 2019-03-04

**Authors:** Francesco Armetta, Maria Luisa Saladino, Cristina Giordano, Chiara Defilippi, Łukasz Marciniak, Dariusz Hreniak, Eugenio Caponetti

**Affiliations:** 1 Dipartimento Scienze e Tecnologie Biologiche, Chimiche e Farmaceutiche - STEBICEF and INSTM UdR - Palermo, Università di Palermo, Parco d’Orleans II, Viale delle Scienze pad. 17, Palermo, I-90128 Italy; 2 School of Biological and Chemical Sciences, Queen Mary University of London, Mile End Road, London, E1 4NS United Kingdom; 3 Stranski-Laboratorium für Physikalische und Theoretische Chemie, Institut für Chemie, Technische Universität Berlin, Straße des 17. Juni 124, Sekr. TC7, D-10623 Berlin, Germany; 4 Institute of Low Temperature and Structure Research, Polish Academy of Sciences, ul. Okolna 2, 50-422 Wrocław, Poland

## Abstract

Ce:YAG nanostructures (Ce:YAG = Cerium in Yttrium Aluminium Garnet), easy to control and shape, have been prepared via templating approach using natural and synthetic materials (i.e. paper, cotton wool and glass wool) previously soaked with a gel-like metals precursor and then thermally treated to achieve the wished morphology. The final material, otherwise difficult to process, can be easily moulded, it is lightweight, portable and forms, at the nanoscale, homogeneous layers of interconnected but not agglomerated nanoparticles (15 ± 5 nm). Using the same synthetic route, called Urea-Glass-Route, but in absence of a template, extremely pure Ce:YAG nanoparticle (45 ± 5 nm) can be also prepared, highly crystalline and well-defined in size and shape. Both structural and optical properties of the final materials were investigated, showing high optical quality. The support allows the production of a multifunctional material with mouldable shape and potential lighting application for large structures combining the strength, chemical durability, fire resistance, and translucency of glass fibres. Last, but not least, the synthetic path also allows an easy scaling up of the process: the first, key step for practical application of nanosized rare-earth doped YAG on large scale.

## Introduction

In the last years the researchers continue to create new materials with tailored and tuneable properties, which enable them to be used in a broad range of applications, including optics, electronics, mechanics, protective coatings, sensors, functional textile fibres, drugs, biomedical materials, and many more. The possibility to prepare mouldable nanostructure directly in one-step process and to model, previously, the shape of the composite could improve the use of luminescent materials for more specific applications^1^.

Biobased, eco-friendly, efficient procedures and economic templates materials have been increasingly considered to give some novel and combined properties^2^. Some examples of hierarchical nanostructures are wood composites, utilized for a range of optoelectronics and energy efficient building materials^3^, or mullite replica of cellulose structures showing luminescence properties for fluorescent probes for sensing and detection as well as biological delivery systems^4^.

In the field of luminescent materials, nanomaterials doped with lanthanide ions have been investigated. Yttrium Aluminium Garnet (YAG, Y_3_Al_5_O_12_) doped with lanthanides ions has received a great deal of attention because can be used for the preparation of nanocomposites useful for the production of white light solid-state LED (Light Emitting Diode), X-ray scintillators^5–7^ and also have great potential in biological applications as long-lasting dye (compared for instance with organic dyes^8–10^).

The production of high-quality Ce:YAG nanostructures is affected by the use of fine, low-agglomerate powders of pure garnet phase because the optical properties of nanocrystals are expected to be dependent on the properties of doping agent and on the synthetic route that may influence particle size and its distribution and morphology^11–13^. Several chemical routes such as co-precipitation^14–19^, sol-gel and Pechini method^7,20–22^, microemulsion method^23–26^, solvothermal synthesis^27,28^ and others have been used to obtain nanopowders. However, most of these methods, unless the solvothermal synthesis, involve pre- or post-synthesis treatments, such as thermal treatments at high temperature, necessary to avoid the formation of secondary phases, e.g. metastable hexagonal yttrium aluminium oxide (YAH), and the monoclinic Y_4_AlO_9_ (YAM) but can also lead to big agglomerated particles, thus leading to a bad efficiency in terms of optical properties. Additionally, these methods require further steps to shape them as nanostructures and nanocomposites, which could affect the optical properties.

In the present paper, the preparation of non-conventional, easy to mould, lightweight and portable Ce:YAG nanostructures is reported. An alternative sol-gel based route (known as the Urea Glass Route, UGR)^29^, previously established for the preparation of metallic nanoceramics, has been here tailored for the synthesis of pure, small yet crystalline Ce:YAG nanoparticles, well-defined in morphology, low degree of aggregation and easily processable for the preparation of nanostructures. The UGR was used thanks to its manifold advantages: it is simple, uses readily available and non-toxic precursors and requires lower reaction temperature compared to the classical ones. Thanks to the formation of an intermediate “gel-/glass-like” material (a complex between the urea and the metal precursor), the primary nanoparticles are stabilized during the heat treatment. In this way, the nanoparticles seeds can nucleate and growth in a controlled way. These nuclei are loaded into a “transient” intermediary organic matrix formed upon decomposition of urea, which converts into volatile product that can leave before the end of the reaction, avoiding post-synthesis purifications, and allowing the preparation of well-defined nanoparticles. Finally, the synthesis is also suitable for large scale production, knowing that a material, although valuable, has no practical applications when produced in small amounts.

The gel-like starting material is also easy to handle and for instance it can be used for soaking a functional support, which, upon thermal treatment, can be directly converted into a tailored nanostructure^30^. Thus, the gel-like precursor was used to embed three structurally different materials here called as “template”, i.e. paper (for integrating the present synthetic path with printing technique), cotton wool (for its natural “fluffy” structure) and glass wool (for its fibers-like structure).

The final structures of all obtained samples have been characterized and the luminescence properties have been determined in order to verify their applicability for lighting applications. These results shown relatively high quantum efficiency for the glass wool templated sample treated at 800 °C, confirming their high luminescence properties.

## Methods

### Materials

Urea (Acros Organics, 98%), ethanol (Aldrich, 99.98%), YCl_3_·6H_2_O (Aldrich, 99.9%), AlCl_3_ (Aldrich, 98%) and CeCl_3_·7H_2_O (Aldrich, 99.99%), sources of Y^3+^, Al^3+^ and Ce^3+^ respectively, were used as received. Solutions were prepared by weight.

### Nanoparticles Preparation

273.9 mg of YCl_3_·6H_2_O, 202.7 mg of AlCl_3_ and 6.8 mg of CeCl_3_·7H_2_O were dissolved in 4 mL of ethanol in order to obtain 2.0% of cerium atoms with respect to total yttrium plus cerium atoms. Proper amounts of urea were then added to the alcoholic salts solution to reach different urea/metals molar ratio (R), according to the route of Giordano *et al*.^31^. A sample containing the metal salts with the same ratio, concentration and solvent, but in absence of urea (R0) has been prepared for comparison.

**Figure 1:**
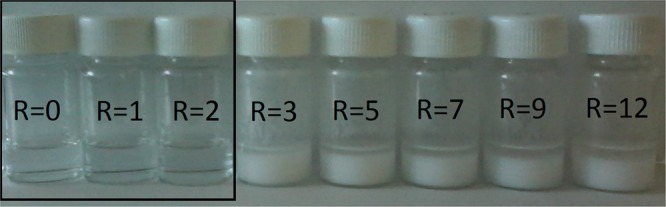
Photo of the starting materials obtained at different urea/metals molar ratio (R). The black square highlights the gel-like systems.

As shown in Fig. 1, for R ≤ 2 the starting materials are clear, homogeneous, colourless and form a colloidal dispersion.

**Figure 2:**
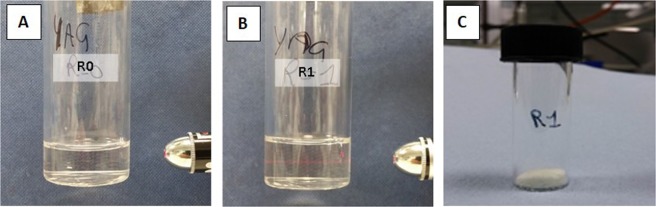
Tyndall effect observed by shining a red laser beam through the R = 1 starting material (**B**), while no effect is observed in the R0 solution (**A**). (**C**) As-prepared powder from sample at R = 1 calcined at 900 °C.

In Fig. 2 it can be nicely observed the Tyndall effect in the R1 sample but not in the R0 using a red laser. For R ≥ 3, the formation of a white dispersion was observed, which separates within few hours. For the purposes of the present paper, only systems at R = 0, 1 and 2 have been further processed. Studies on the nature of these systems (R > 2), alongside a more detailed study on the nature of the starting gels (R ≤  2), i.e. the coordination shell around the metals in presence of urea, are currently in progress. The removal of the solvent via drying process brings to neither the crystal salts, nor to the appearance of pure urea, rather to a white gum-like compound highly hygroscopic, indirectly confirming the urea-metal(s) complex formation.

After preparation, samples at R < 2 were calcined up to 900 °C (heating ramp 6 °C/min). Thermal treatments at intermediate temperatures (300, 500, 700 and 800 °C) were also performed to study the formation mechanism of the garnet phase. After reaching the final temperature and allowing 1 h dwelling time, the samples were extracted from the oven and let cool down. The final material is a yellowish solid in form of small pellets, which were grounded in a mortar to obtain a fine powder (Fig. 2C).

### Nanostructures Preparation

**Figure 3:**
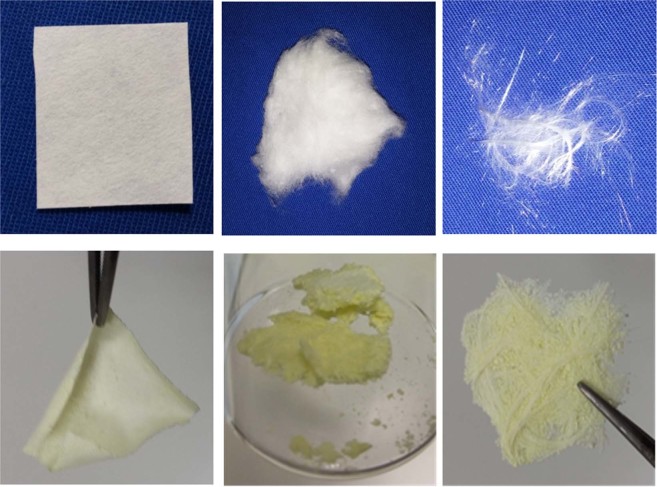
Photos of the template before calcination (up) and after calcination (previously soaked with the YAG gel-like precursors, down). From left to right: filter paper, cotton wool and glass wool, respectively.

Filter paper (Whatman), cotton wool (HypaCover extrafine) and glass wool (Assistent) were chosen as templates. They were imbued with the starting gel-like material at R = 1 and treated at 800 °C or 900 °C for 1 hour each. As previously observed for the case of metallic nanoceramics^30^, also in the present case the macroscopic original shape of the template is remarkably preserved (Fig. 3). As expected, calcining the templates in absence of the YAG precursors, the template loose its shape (see Figs SI1 and SI2 of the Support Information).

### Characterization techniques

Thermo-gravimetric analysis (TGA) was performed with a Q500 TA instrument under an air flow of 60 cm^3^ min^-1^ for the sample and a nitrogen flow of 40 cm^3^ min^−1^ for the balance. The weight of each sample was ca. 40 mg. The measurements were carried out by heating the sample from room temperature to 900 °C at a rate of 6 °C min^−1^. The powder X-Ray Diffraction (PXRD) patterns were recorded with a Panalytical Empyrean Diffractometer (Cu Kα radiation, λ = 0.154 nm). Powder patterns were analysed according to the Rietveld method^32^ using the MAUD software^33^. N_2_ adsorption-desorption isotherms were registered at 77 K using a Quantachrome Nova 2200 Multi-Station High Speed Gas Sorption Analyzer. Samples were outgassed for 12 h at room temperature in the degas station. Adsorbed nitrogen volumes were normalized to the standard temperature and pressure. The specific surface area (S_BET_) was calculated according to the standard BET method (based on the Brunauer–Emmett–Teller theory) in the relative absorption pressure (P/P_0_) range from 0.045 to 0.250^34^. Attenuated Total Reflection (ATR) Infrared Spectra were acquired between 600 and 4000 cm^−1^ by using a Perkin Elmer Spectrum 65 Spectrometer equipped with an ATR setup. Scanning Electron Microscopy (SEM) images were acquired on a FEI inspect F instrument. The samples were loaded on carbon coated stubs and coated by sputtering an Au alloy prior to imaging. Transmission Electron Microscopy (TEM) micrographs were acquired by using a JEOL 2010 electron microscope operating at 200 kV accelerating voltage, equipped with an energy dispersive X-ray spectrometer (EDS, Oxford, UK) suitable for element identification. Samples were ground and then suspended in ethanol. One drop of this suspension was put on a holey-carbon coated copper grid of 300 mesh and left to air to dry. Emission and excitation spectra were measured using the FLS980 Fluorescence Spectrometer from Edinburgh Instruments equipped with 450 W Xenon lamp. Both the excitation and emission 300 mm focal length monochromators were in Czerny Turner configuration. Excitation arm was supplied with holographic grating of 1800 lines/mm, blazed at 250 nm, while the emission spectra was supplied with ruled grating, 1800 lines/mm blazed at 500 nm. The spectral resolution was 0.1 nm. The R928P side window photomultiplier tube from Hamamatsu was used as a detector. Quantum efficiency of luminescence was measured using the same equipment supplied with 30 cm in diameter integrating sphere. The lifetime measurements were carried on a femtosecond laser setup composed of a Coherent LibraS all-in-one ultrafast oscillator and regenerative amplifier laser system, with pulse duration less than 100 fs at 1 kHz repetition rate, a Coherent OPerASolo optical parametric amplifier and a Hamamatsu C5677 streak camera with time resolution of 14 ps.

## Discussion of Results

### Nanoparticles preparation

**Figure 4:**
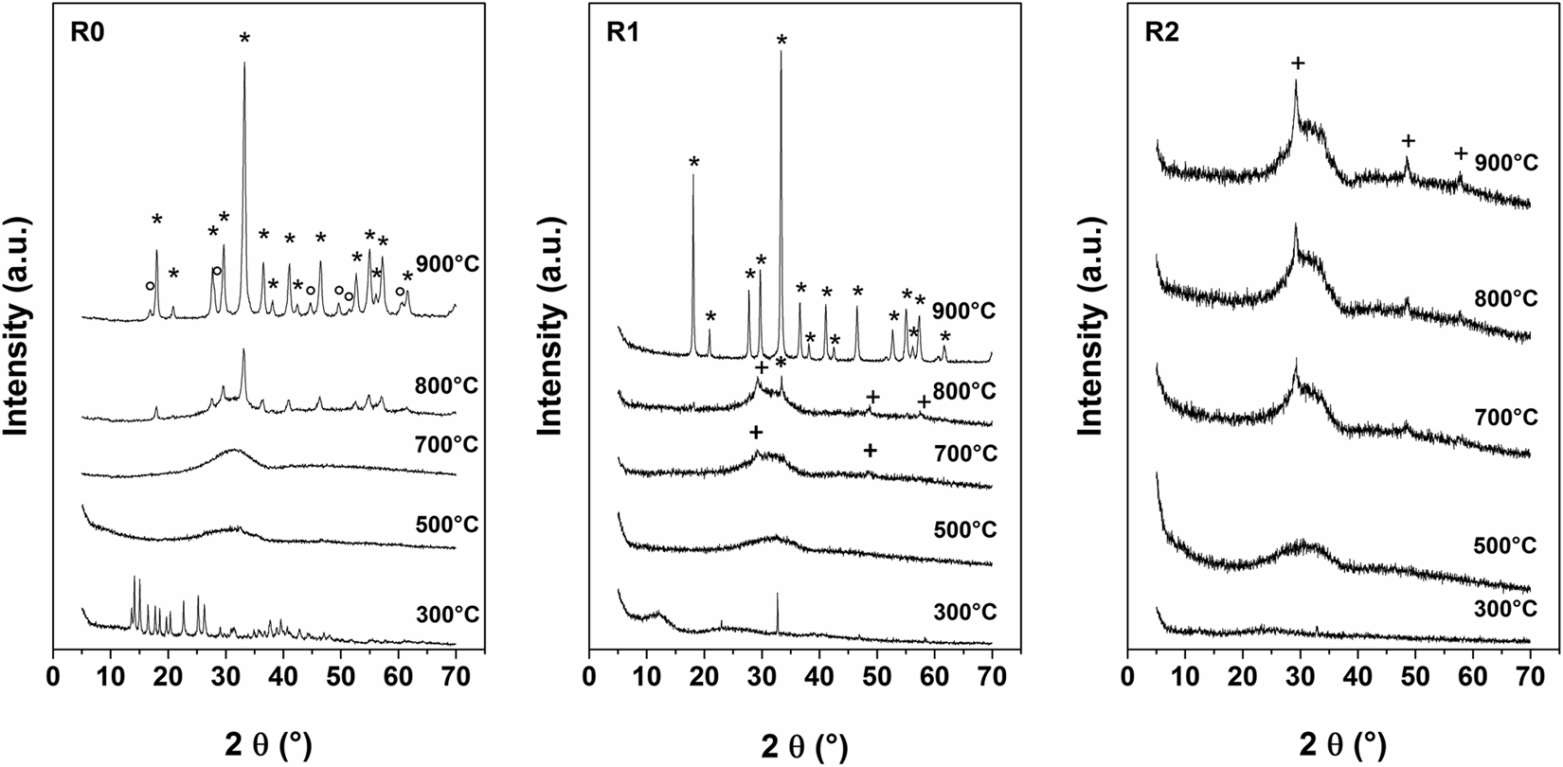
XRD patterns of the powder obtained by thermal treatment of starting systems at R = 0, 1 and 2. Phases present at highest temperatures: *YAG, °YAH and ^+^Yttria.

The gel-like starting material at R ≤  2 (see Fig. 1) have been calcined at temperatures between 300 and 900 °C. The corresponding powders, made without the use of any template, have been characterized via PXRD. The corresponding patterns are shown in Fig. 4.

In the physical mixture of the metal salts prepared without addition of urea (R0), the crystalline phase observed after the treatment at 300 °C can be attributed to the mixture of reactants, YCl_3_·6H_2_O and AlCl_3_ (see Fig. SI3 of S.I.), while only an amorphous phase can be observed from 500 °C to 700 °C. The formation of the crystalline garnet phase can be observed in the sample treated at 800 and 900 °C, alongside a large amount of an amorphous phase (55%) and the hexagonal phase of yttrium aluminium oxide (YAH, 8.9%), respectively, according to the Rietveld refinement. For samples prepared with an initial ratio R = 1 an amorphous phase is mainly observed up to 700 °C. At 700 °C, the two small peaks at 29.1° and 48.5°, superimposed to the amorphous, can be ascribed to the (222) and (440) planes of the cubic yttrium oxide (Y_2_O_3_, yttria) phase, while the XRD pattern of the system treated at 800 °C shows an increase of these peaks, alongside the appearance of the YAG main peak at ~33°. The pure garnet phase is observed at 900 °C and no traces of other species are present. For the sample at R = 2, mainly an amorphous phase can be observed at any temperature, with traces of yttria observed for T > 700 °C. The intensities of these peaks increase with increasing T.

This finding indicates that for both samples (regardless of R) the formation of YAG does not start from a mere mixture of metal salts or hydrated salt, but follows the formation of the intermediate glass-like phase previously observed within the urea-glass-route^31^. The ability of urea to polymerise and to form complexes with transition metals is well-known. The bonding occurs either via bridging the metal with urea carbonyl group or the amide groups^35^. We attributed the gel-like appearance of our starting materials with the metal-urea complexes formation. During the heat treatment, an intermediate organic matrix is formed from the decomposition of urea^36^, where the metal ions are loaded onto. During calcination, alongside the matrix decomposition, the nanoparticles start to nuclei and growth and, upon further increase of the temperature, crystallize. By the end of the heat treatment the matrix has left. Noteworthy, the decomposition of the organic matrix (mainly constituted by C and N), creates a local reducing atmosphere, which slow down the formation of oxides. The assistance of urea in creating a reducing atmosphere during the YAG formation is somehow confirmed by the XRD patterns of sample prepared at R = 2, where the formation of the YAG phase cannot be observed even at T = 900 °C (only traces of yttria can be seen). On the other end, in agreement with the IR data, these findings evidence the role of urea, which probably partially bond the aluminium and affect its availability to participate to the crystallization process, involving the formation of yttria, as previously reported in the case of YAG nanoparticles prepared in microemulsion^25,26^.

The Rietveld refinement^32^ has been applied to the XRD pattern of the samples treated at 800 and 900 °C by using the MAUD software^33^. The Rietveld best fit reproduces well the diffraction pattern of each sample. As an example, the XRD pattern, the correspondent Rietveld fit and the residual plot of the sample R1 treated at 900 °C are shown in the Fig. SI4 of S.I. The percentage of amorphous phase was determined by using the method proposed by Brindley *et al*.^37,38^. The results of the quantitative phase determination together with the cell parameter for each phase, crystallite size and lattice microstrain are reported in Table SI.I of the S.I. The value of cell parameter of the garnet phase obtained from the starting system R = 0 treated at 800 and 900 °C and R = 1 at 900 °C is greater than the characteristic one (a = 12.016 Å), indicating that the cerium ions have substituted the yttrium ions in the lattice. The presence of appreciable line broadening, also evaluated within the Rietveld refinement, is ascribable to small crystallite size D and to the presence of lattice microstrain.

### Nanostructures characterization

**Figure 5:**
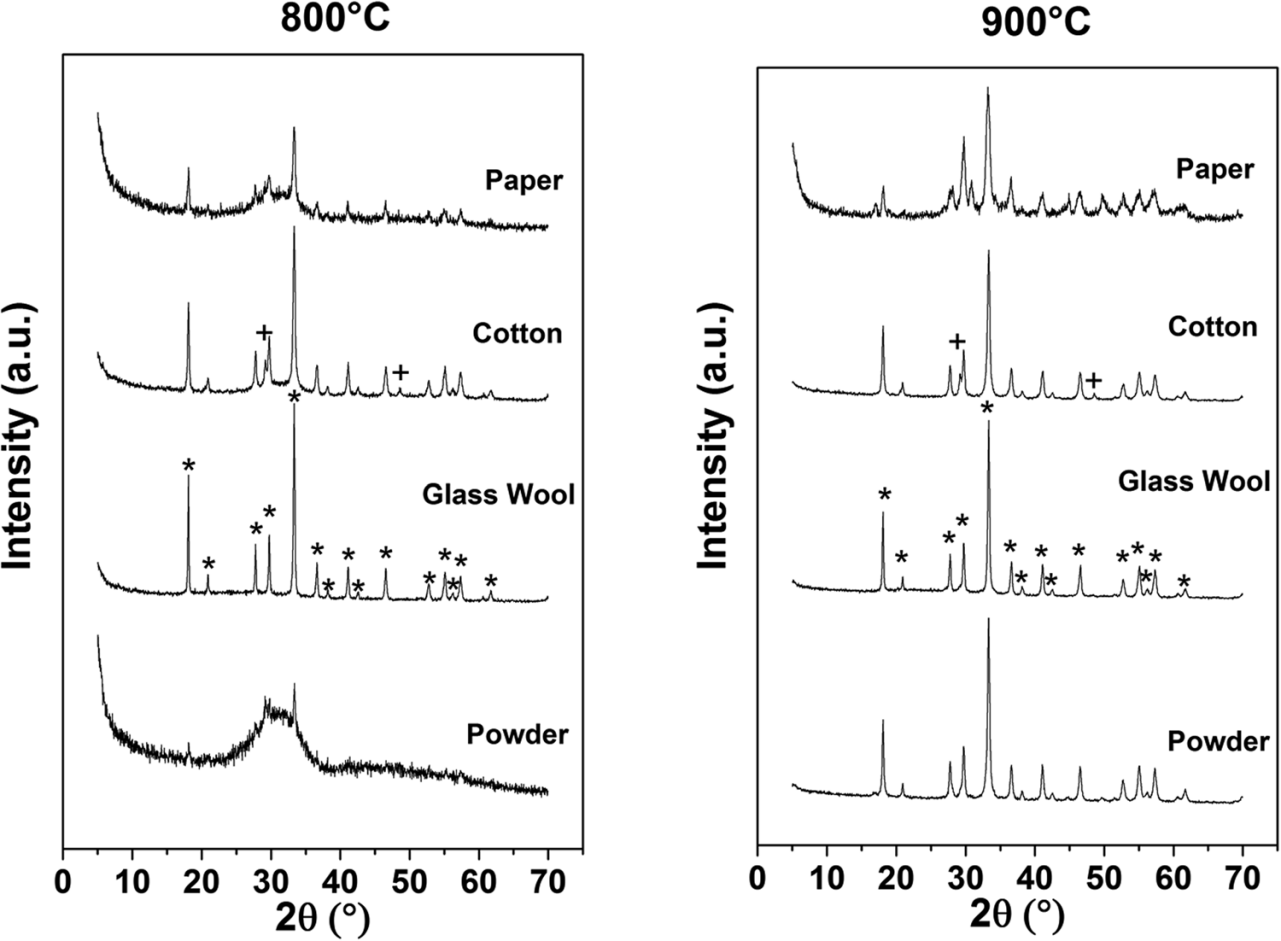
XRD patterns of the powders and of the templated samples obtained by calcination at 800 °C and 900 °C with R = 1. *YAG, and ^+^yttria.

Considering the results obtained for the preparation of pure YAG nanoparticles, same reaction conditions has been used also for the preparation of the YAG-based nanostructures. The gel-like starting material prepared with R = 1 was used to embed three structurally different materials, namely paper, cotton wool and glass wool. For simplicity we will refer to these systems as “templated samples” (or using the name of the corresponding template) and will indicate as “powder” the sample prepared without any template. After soaking, the samples were calcined at 800 and 900 °C for 1 h each and the corresponding XRD patterns are reported in Fig. 5. For comparison, the XRD pattern of the sample prepared in the same conditions without any template (at R = 1) is also reported.

Differently than for the powder, in all samples prepared in the presence of a template, crystalline phase(s) can be observed at both temperatures. However, only the XRD patterns of the glass wool template samples, at both temperatures, display the alone crystalline garnet phase. This finding is also unexpected because the powders obtained at 800 °C is constituted by a mixture of garnet and yttria phase and amorphous. Probably, the by-product generated by the decomposition of cotton and paper interact with the aluminum and yttrium ions influencing the formation of a nanostructure where the single YAG phase is present. This is surely due to the different chemical composition of the template material, where cotton (a cellulose based matrix) has a more complex structure than glass wool, where basically just silicon and oxygen are present. Since a deeper investigation on the influence of template structure over the final composition of the YAG nanostructures deserves specific studies, out of the scope of this work, we prefer to postpone this investigation to a later paper.

The results of Rietveld refinement to all XRD patterns of the templated samples are reported in Table SI.II of the S.I. At both temperatures, a mixture of different phases, more or less amorphous, can be observed in the case of cotton and paper. At 800 °C, the YAG phase is 100%, 82% and 21% for glass wool, cotton and paper, respectively. At 900 °C, the percentage of garnet phase has increased in the cotton and paper samples, but the amorphous phase is still present together with yttria as side product.

As for the powders, also in the glass wool template sample, the value of cell parameter of the garnet is greater than the characteristic one (a = 12.016 Å), indicating that the cerium ions have substituted the yttrium ions in the lattice. The crystallite size in the glass wool template sample is similar to the ones in the powder after the calcination at the same temperature, while the lattice microstrain is higher in the glass wool template sample obtained at 900 °C indicating a more distorted garnet structure.

Nitrogen adsorption-desorption isotherms were obtained to gather information on the specific surface area (S_BET_). The S_BET_ was 36.6 ± 0.1 m^2^ g^−1^, one order of magnitude greater than that of the same material obtained by co-precipitation method (3.15 ± 0.06 m^2^ g^−1^) confirming that particles are smaller and/or their degree of aggregation is lower. Similar value has been obtained for nanoparticles prepared in microemulsion^23^. For the glass wool templated samples obtained at 800 and 900 °C, the S_BET_ values are 9.36 ± 0.5 and 8.55 ± 0.4 m^2^ g^−1^, respectively. Both values are lower respect to the powder and very close to the S_BET_ of the glass wool (3.84 ± 0.6 m^2^ g^−1^) indicating that the two materials are similar to the glass wool used as support.

**Figure 6:**
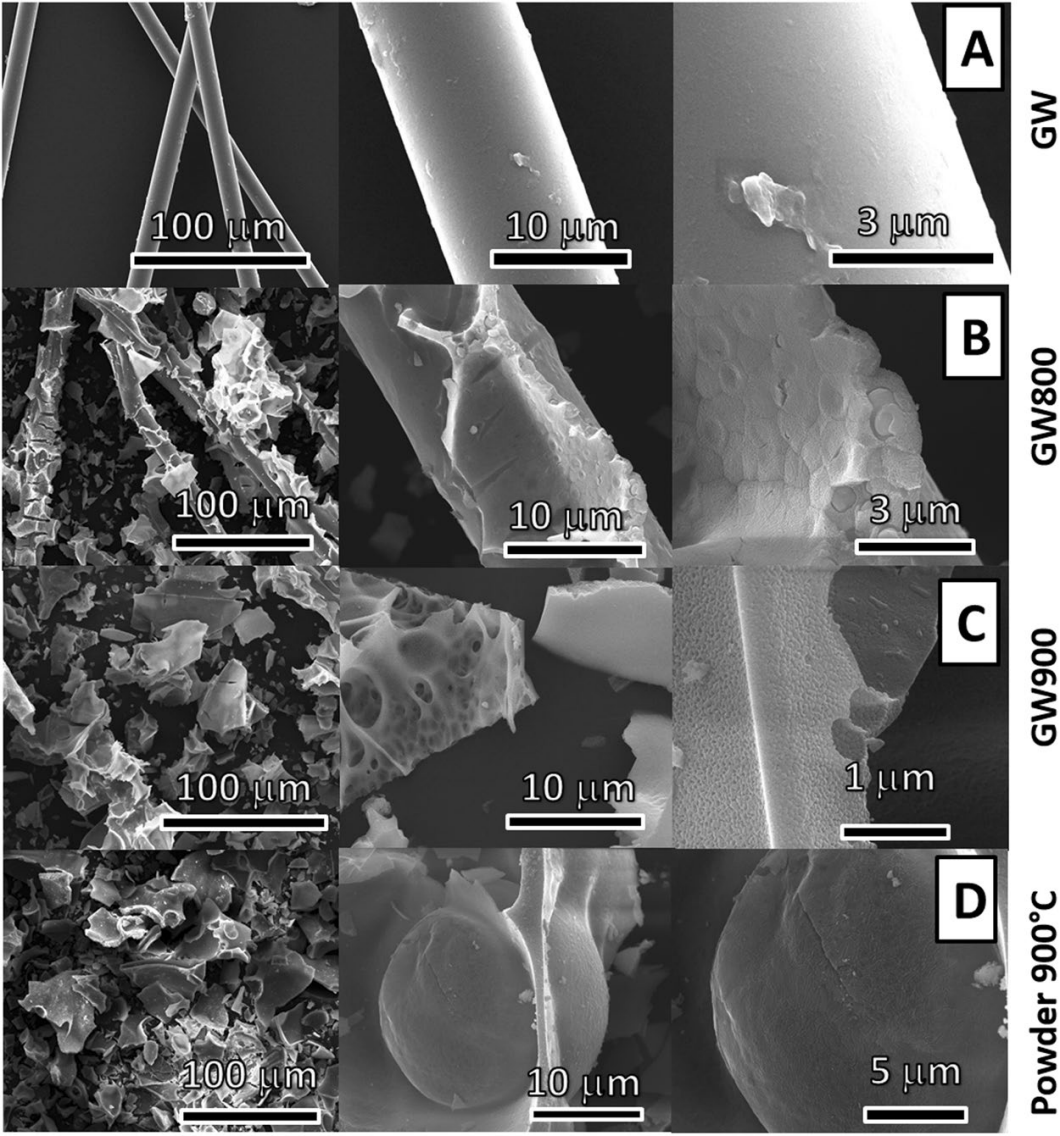
SEM micrographs of (**A**) glass wool as template, glass wool templated samples treated at (**B**) 800 °C and (**C**) 900 °C, (**D**) powders obtained at 900 °C.

Optical and electron microscopy studies were performed in order to investigate the morphology and to evaluate the particle size both in the as-prepared powder and in the glass wool templated samples. Optical microscope images of glass wool and of glass wool template samples obtained at 800 °C and 900 °C are reported in Fig. SI5 of the S.I. The images show that the glass wool templated samples obtained at 800 °C is similar to the original glass wool (before calcination) even if the fibres are shorter and covered by particles. SEM micrographs at different magnifications of the glass wool templated samples are reported in Fig. 6.

SEM micrographs of the original glass wool and of the powder are also shown. It is worth to note that the original glass wool shows a very smooth surface (Fig. 6A) and that, after the calcination at 800 °C, the fibres are melted and coalesced (Figs SI1 and SI2 of the S.I.). On the contrary, the templated sample obtained at 800 °C had inherited the backbone of the original glass wool, now converted however into a closely packed, well-structured and homogeneous layer of nanoparticles. The presence of “craters” on the structure of the templated sample (visible in Fig. 6B at higher magnification) clearly shows that the nanoparticles are not just lying on the surface but are part of the whole structure. The nanoparticles in the templated sample are plate-like but of more defined sizes, ranging from 20 to 40 nm. The templated sample obtained at 900 °C (Fig. 6C) did not maintain the fibrous appearance of glass wool. Blocks from coalesced fibres are observed; also, unorganized clusters of nanoparticles, mainly plate-like and with sizes ranging from 20 to 50 nm are present, similar to the ones observed in the powder (Fig. 6D). The local composition of the obtained templated samples were investigated by EDS. The X-ray fluorescence spectra (not shown), acquired for some nanoparticles, show the peaks of yttrium, aluminium and cerium present in the samples, including calcium, potassium, sodium, magnesium, and silicon of the glass wool.

**Figure 7:**
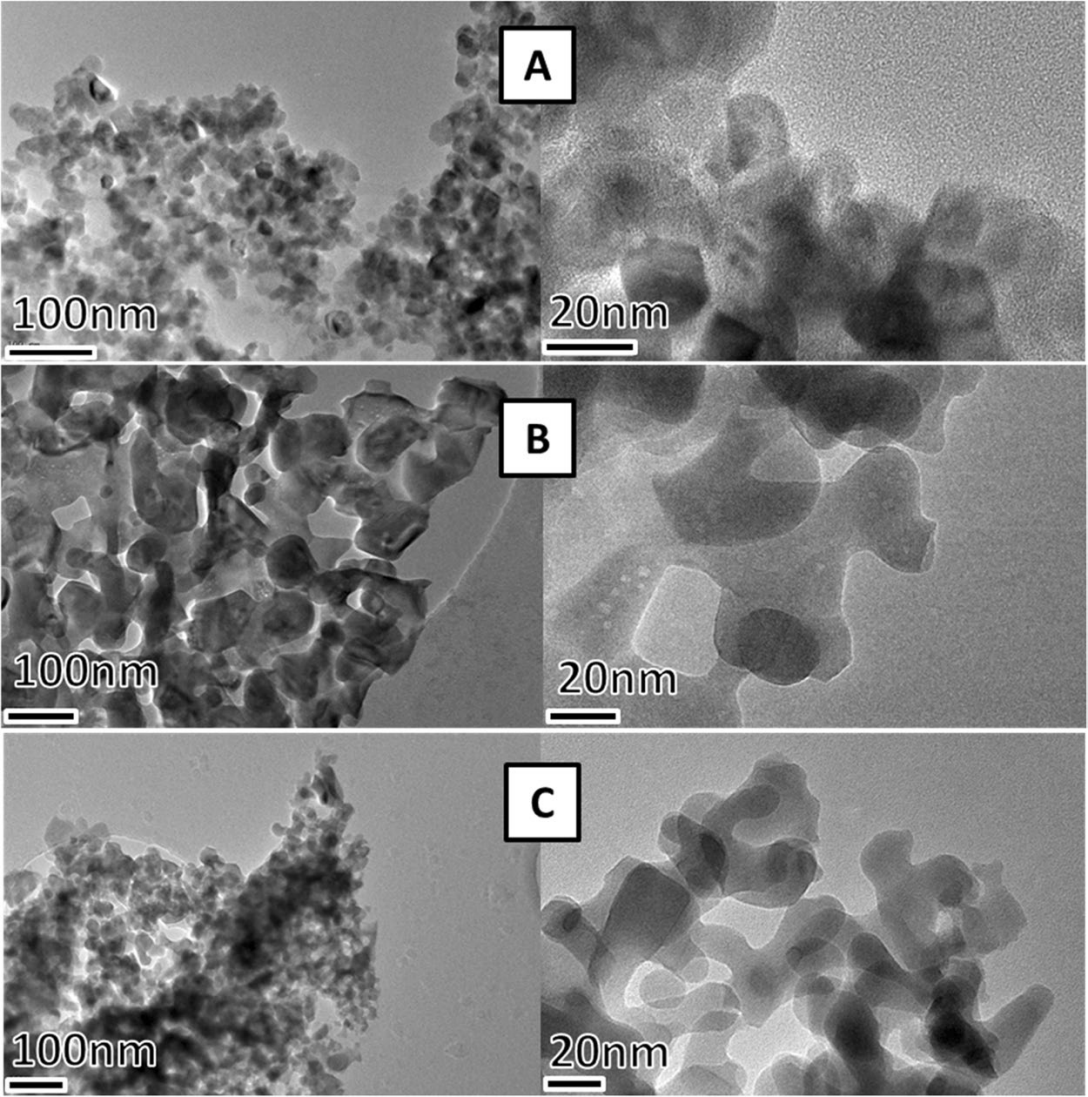
TEM micrographs of glass wool templated samples treated at (**A**) 800 °C and (**B**) 900 °C and of (**C**) powder obtained at 900 °C.

To have a better insight of the nanoparticles size and shape, TEM investigation has been performed. Micrographs of the powder and templated samples acquired at different magnifications are shown in Fig. 7. The glass wool sample treated at 800 °C presents homogeneous clusters of interconnected nanoparticles (15 ± 5 nm). At 900 °C bigger, less defined in shape nanoparticles can be observed. TEM confirms the plate-like shape of the nanoparticles both in the powder (45 ± 5 nm) and the templated sample (17 ± 5 nm) treated at 900 °C. The particle size distributions are reported in Fig. SI6 of S.I.

### Characterization of the gel-like starting materials and study on the nanoparticles formation mechanism

**Figure 8:**
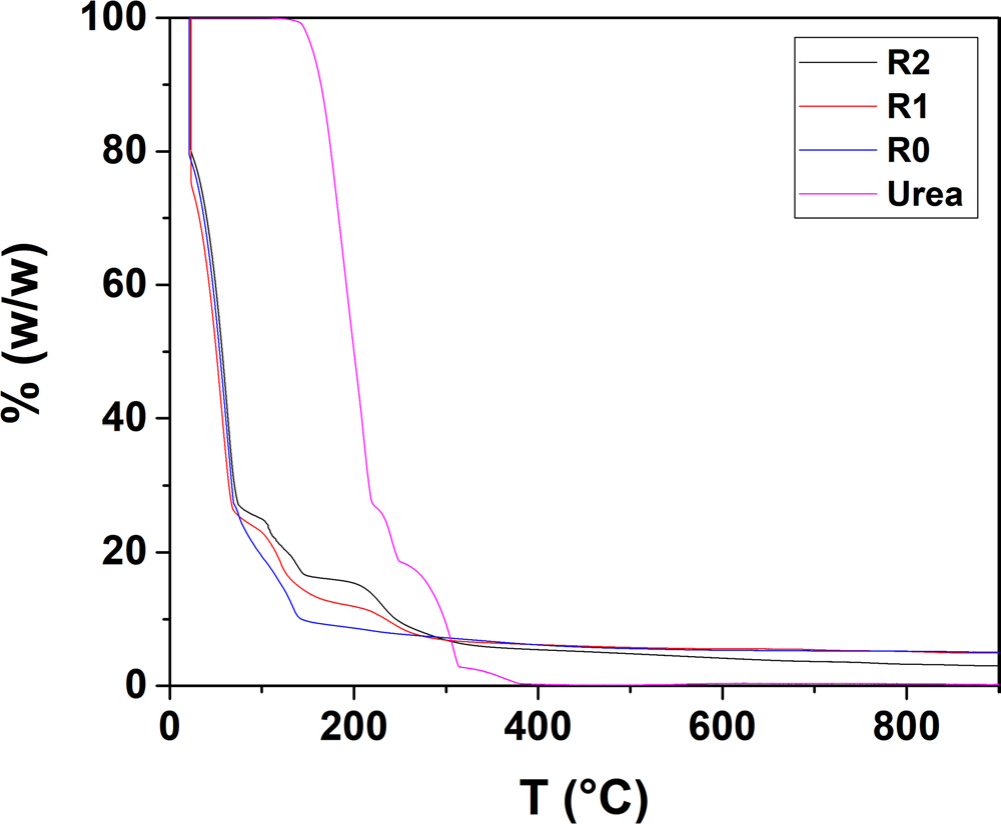
TGA patterns of the starting gel-like materials prepared with R = 0, 1 and 2.

The thermogravimetric analysis (TGA) result of the starting systems prepared with different R is reported in Fig. 8. For comparison and discussion purposes, the TGA curve of pure urea has also been included.

The TGA pattern reveals interesting discussion points. The dissimilarities between the decomposition patterns of pure urea and the sample prepared in absence of urea (R = 0), with those of samples at R = 1 and 2, confirm the formation of urea-metal complexes. For pure urea (pattern recorded on the solid phase), decomposition starts at T ~ 200 °C and it is completed below 400 °C as expected^36^. According to the work of Schaber *et al*., for the decomposition of pure urea under air, three main steps are expected, involving the release of ammonia (up to T ~ 250 °C) and condensation reactions with formation of bigger molecules (including meleme and melone, up to T ~ 300 °C), which upon further temperature increase (T > 300 °C), decompose releasing mainly CO_x_^36^. For the R = 0 sample, after an initial solvent release (below 100 °C) the final product is formed already at T ~ 150 °C and further temperatures increase might just assist the crystallization, as previously observed in similar studies^15,39^. For the complexes (R = 1 and R = 2), in both cases after an initial solvent release (below 100 °C), samples decomposition takes place and it is completed below 300 °C. However, the smoother decomposition of sample R = 1 is similar to that previously observed for urea-metal complexes^31^, while R = 2 decomposes in sharper steps, closer to those observed in the pure urea. This finding leads to think that only part of the urea is coordinated to the metal(s), while the rest is possibly allocating in an outer shell. More interesting, the complexes decomposition starts and it is completed at lower temperature compared to pure urea, which is opposite to what observed for other urea-metal complexes^31^. Together with the IR results reported below we believe that this behaviour is due to the coordination of the central metal ion through the amine groups of urea (rather than via oxygen bridges as previously observed)^40^, which would “break” the hydrogen bonding naturally presents in the structure of pure urea, and thus “destructuring” the metal-urea network, which decomposes at lower temperature (compared to pure urea).

**Figure 9:**
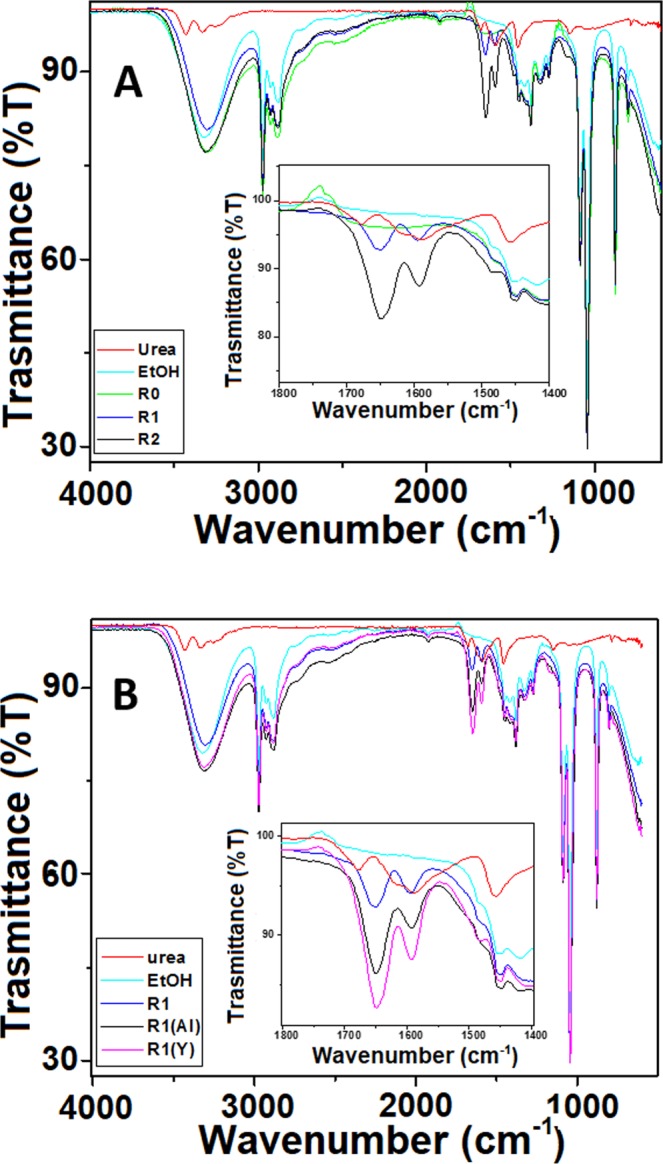
IR spectra of (**A**) the starting systems at R = 0, 1 and 2 and (**B**) of system at R = 1 containing alone yttrium and aluminium ions.

From the IR study it was observed that the yttrium metal ions could play a major role in binding with urea molecules. In Fig. 9, the starting complex prepared at R = 1 and the sample prepared using aluminium and yttrium salts separately (respecting the total concentration used in the mixture) are shown. The IR spectra of pure urea and ethanol are also reported for comparison. As it can be seen from Fig. 9, the spectra are mainly dominated by the presence of ethanol, which seems to play the only role of solvating agent and does not take part to the coordination shell of the complex, as observed for instance for other urea metal complexes^31^. The urea carbonyl region (1650–1550 cm^−1^) however is free from solvent peaks and some considerations can be made. In this region for pure urea two bands can be observed, one centred at 1678 cm^−1^ and the other one at 1588 cm^−1^ (with a shoulder at 1616 cm^−1^), attributed to the to the symmetric bending of -NH_2_ and to the stretching of the C=O group (with the contribution of -NH_2_ asymmetric bending, as shoulder), respectively^41^.

In the IR spectrum of the sample prepared at R = 1, the small shoulder (due to the contribution of the -NH_2_ asymmetric bending) disappears and the C=O peak become sharper, slightly shifting to higher wavenumber (1595 cm^−1^ ← 1588 cm^−1^). More interesting, the intensity of the peak of the symmetric bending of NH_2_ increases appreciably and shifts to lower wavenumber (1650 cm^−1^ ← 1678 cm^−1^). Although, both band shape and peaks position are different from those in the pure urea, they seem to be affected neither by R nor by the number of metal ions present in the complex (see Table SI.III in S.I.). On the other hand, the intensity of both peaks increases with increasing R and, more interesting, the ratio between the -NH_2_ and the CO peaks increase in the complexes (regardless of R).

On what concern the comparison between the spectra of sample R1 (which includes all metallic elements) and samples prepared with only one metal ion (Y^3+^ or Al^3+^ respectively) similar considerations can be done. In the monometallic samples, the carbonyl region shows two bands, centred at ~1650 cm^−1^ and ~1590 cm^−1^ in both cases, while the intensity and ratio of the -NH_2_ and the CO peak is very different, with a more intense -NH_2_ band.

Based on these findings, we assume that the presence of the metal ions might disrupt the H-bonds present in the urea network^42^, and partially bond with the NH_2_ (possibly via electrostatic and dipole-dipole interactions). This seems to be confirmed by the disappearance of the δ_as_(NH_2_) contribution (as a shoulder in the CO peak) and the increase of the intensity for the δ_s_(NH_2_), which would indicate a difference in the dipole moment following the vibration. The presence of the metal ions close to the -NH_2_ groups might also shift the electron clouds on the N atoms, which would be slightly pauperize the C and come closer to the electron rich oxygen. The shift of CO band to higher wavenumber compared to pure urea (i.e. a stiffer C=O bond) seems to support this finding and excludes metal-urea bonds via oxygen bridges (the presence of the metal ion close to the oxygen would weaken and lengthen the C=O bond).

### Optical Properties

**Figure 10:**
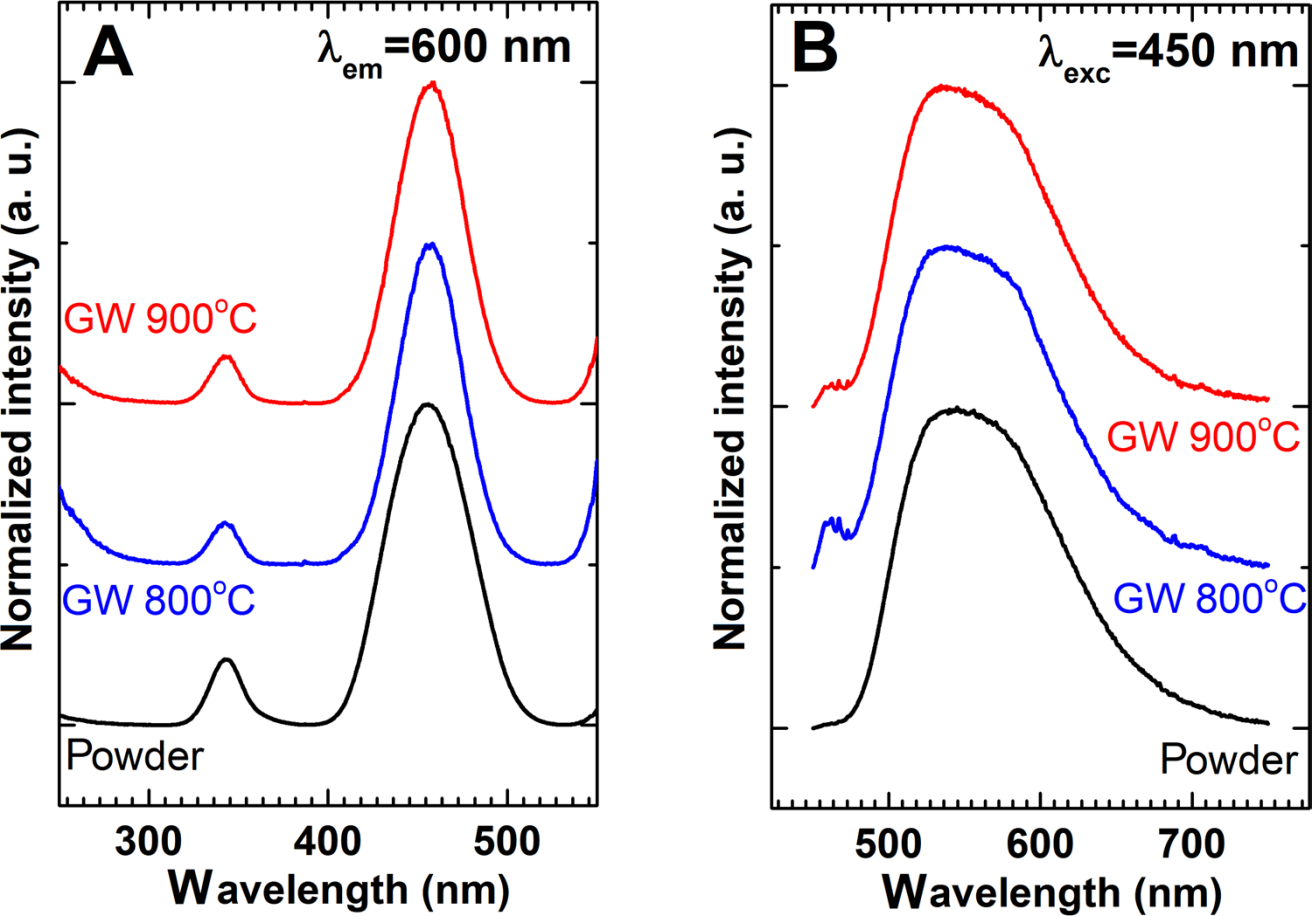
Excitation (**A**) and emission (**B**) spectra of powder obtained by calcinations at 900 °C of starting system at R = 1, and of the glass wool templated samples obtained at 800 °C and 900 °C.

In order to verify how the optical properties of the glass wool templated sample are affected by the presented preparation procedure, a detailed spectroscopic analysis was performed. The excitation and emission spectra of the powder, obtained by calcinations at 900 °C of the starting system at R = 1, and of the glass wool templated sample obtained at 800 °C and 900 °C are reported in Fig. 10.

The excitation spectra of the obtained powders and nanostructures consist of two characteristic for Ce^3+^ ions absorption bands centred at 340 nm and 460 nm assigned to ^2^F_5/2_ → 5d^1^ electronic transition. Although positions of the bands are independent on the preparation procedure, a slight difference in relative bands’ intensities can be found. The intensity of 340 nm band decreases slightly in respect to the 460 nm band for the nanocomposites comparing to the Ce:YAG powder. This effect is related to the energy losses associated with the scattering of the UV light by the glass wool and suggest that 460 nm absorption band is more suitable for the excitation of the luminescence. Emission spectra of obtained materials upon 450 nm excitation presented in Fig. 10B reveal the presence of inhomogeneously broadened emission that consists of two bands related with 5d → ^2^F_5/2_ and 5d → ^2^F_7/2_ electronic transition of Ce^3+^ ions. No significant difference can be found in the shape of emission spectra, what confirms good luminescent properties of prepared glass wool templated sample.

**Figure 11:**
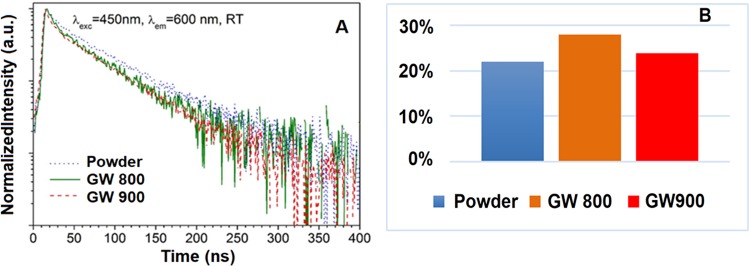
Room temperature luminescence decay profiles of Ce^3+^ ions (**A**) and quantum efficiencies (**B**) of powder obtained by calcination at 900 °C of starting system at R = 1, and of the glass wool templated samples obtained at 800 °C and 900 °C.

The single exponential decay dominates in the kinetic profiles presented in Fig. 11A. The fast component observed at the beginning of the profile is probably related to the short-leaving excited states of the Ce^3+^ ions located at the strongly defected surface part of the nanocrystal. However, since the contribution of this component to the total decay is minor to the total decay profile, we have decided to use exponential function to fit experimental data.

The analysis of the kinetics of excited state of Ce^3+^ ions reveals that the decay profile of the glass wool templated samples are similar to the one obtained for the powder (decay times for both GW samples are decreased from 50 μs by approximately 10%) what confirms that preparation procedure does not provide any significant channels of nonradiative depopulation of 5d^1^ state.

As it can be seen in Fig. 11B, the quantum efficiency is the highest for the glass wool sample treated at 800 °C and reaches around 27%. This is still significantly reduced compared to the commercially available Ce:YAG microsized phosphor characterized by three times higher value^43^. Observed decrease of the value is mainly due to the nonradiative depopulation processes of the excited states related with the large amount of the Ce^3+^ ions is localized in the strongly defected surface area of the nanoparticle^44,45^ that is confirmed also by the presence of short component in the decay of Ce:YAG nanocomposites (Fig. 11A). On the other hand, it is observed a relative decrease of quantum efficiency for all glass wool templated samples treated at 900 °C.

It may be explained by higher scattering and absorption observed both for powders and shapeless nanocomposite samples in comparison to the glass wool templated samples treated at 800 °C. Additionally, the microstrains, exemplifying qualitatively structural deformations of the YAG structure (Table SI.II of S.I), produced most probably as a result of the collapsing of wool glass microstructure, also reached their maximum for the glass wool sample treated at 900 °C. Summarizing, quantum efficiency recorded for the glass wool templated sample treated at 800 °C confirms their high luminescence properties, particularly for the heavier doped samples, for which the recorded quantum efficiency is also higher than for the corresponding Ce:YAG nanopowders.

## Conclusions

An unique synthetic pathway for the preparation of Ce:YAG nanostructures has been presented. The synthesis involves the assistance of readily available objects embedded with a suitable gel-like material. The new synthetic pathway is not expensive, easily scalable for large scale production and open up the way to the preparation of tailored optical devices. In general, the possibility to convert easily adaptable materials (e.g. foldable paper or unrolling glass wool) into organized, still highly functional, doped YAG nanoparticles, opens up the way to a myriad of possibility to prepare systems that can be easily shaped and then lighted up.

The gel-like precursor (metals/urea complex) ensures the formation of highly pure and crystalline garnet phase, while the template allows its preparation as well-structured, homogeneous and compact layers of nanoparticles having good optical properties. In addition, the prepared structure could combine the optical properties with the properties of the original template. Knowing that special glasses have been developed with low dielectric constant to serve in certain radar installations, with high lead content for radiation shielding, and with exceptionally high strength and modulus of elasticity for critical reinforced plastics applications, the unique properties of the luminescent glass wool nanostructure have great potential in applications for large structures including lighting applications combining the strength, chemical durability, fire resistance, and translucency of glass fibres.

## Supplementary information

Supplementary info
