# Supplementary material for: Non-conventional Ce:YAG nanostructures via urea complexes
Source: Sci Rep. 2019 Mar 4;9:3368. doi: 10.1038/s41598-019-39069-6 (PMC6399219; doi:10.1038/s41598-019-39069-6)
Supplement: Supplementary file 1 — Supplementary info [file 41598_2019_39069_MOESM1_ESM.docx]

Support Information

**Non-conventional Ce:YAG nanostructures via urea complexes**

**Francesco Armetta**1**, Maria Luisa Saladino**1,* **Cristina Giordano**2,3,***, Chiara Defilippi**2**, Łukasz Marciniak**4**, Dariusz Hreniak**4**, Eugenio Caponetti**1

1 Dipartimento Scienze e Tecnologie Biologiche, Chimiche e Farmaceutiche - STEBICEF and INSTM UdR - Palermo, Università di Palermo, Parco d’Orleans II, Viale delle Scienze pad.17, Palermo I-90128, Italy

2 Centro Grandi Apparecchiature-ATeN Center, Università di Palermo, Via F. Marini 14, Palermo I-90128, Italy

3 School of Biological and Chemical Sciences, Queen Mary University of London, Mile End Road, London E1 4NS, United Kingdom

4 Stranski-Laboratorium für Physikalische und Theoretische Chemie, Institut für Chemie, Technische Universität Berlin, Straße des 17. Juni 124, Sekr. TC7, D-10623 Berlin, Germany

5 Institute of Low Temperature and Structure Research, Polish Academy of Sciences, ul. Okolna 2, 50-422 Wrocław, Poland

**
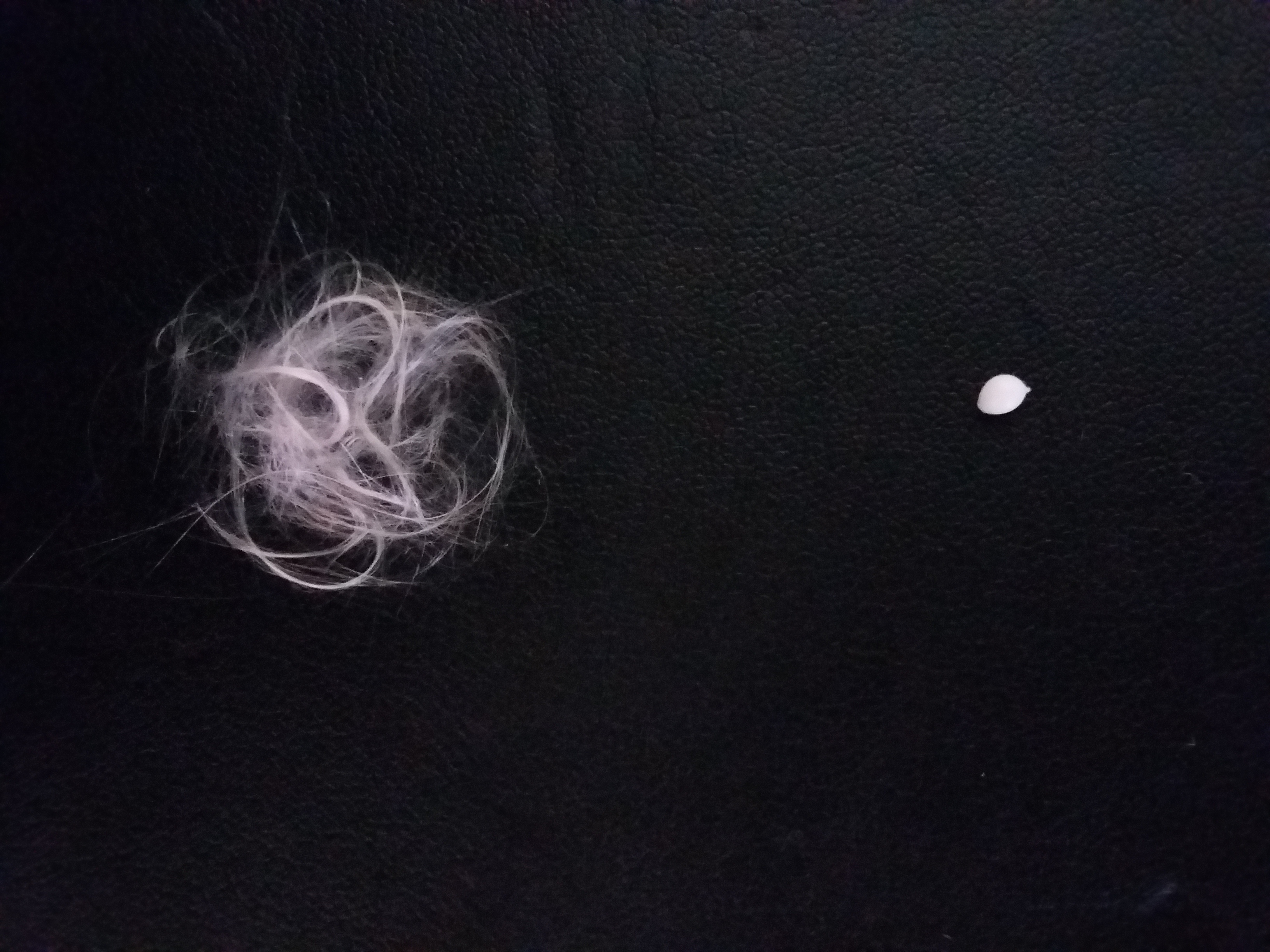
**

Figure SI1. Photos of the glass wool before (left) and after (right) calcination at 800°C.

**
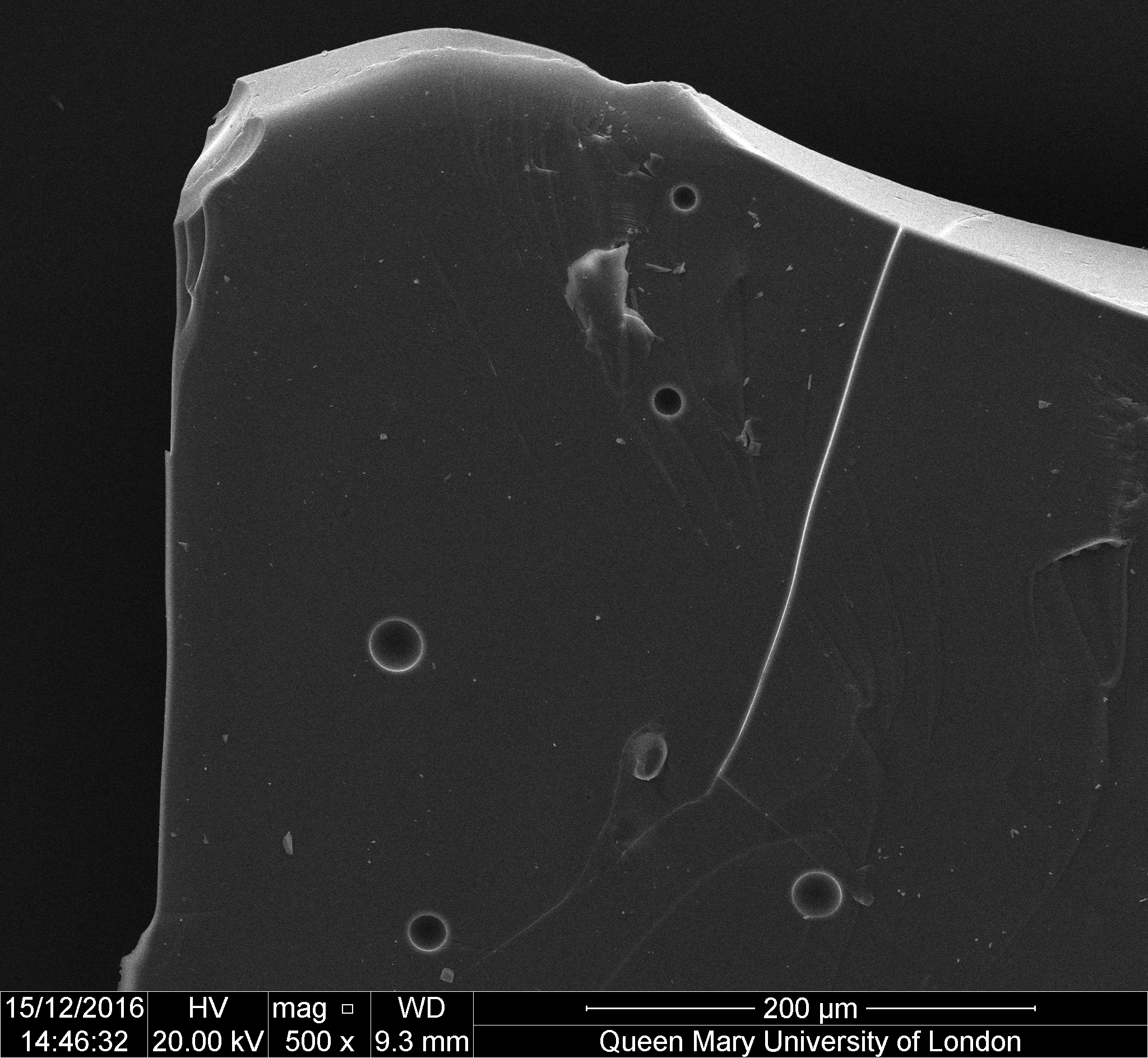

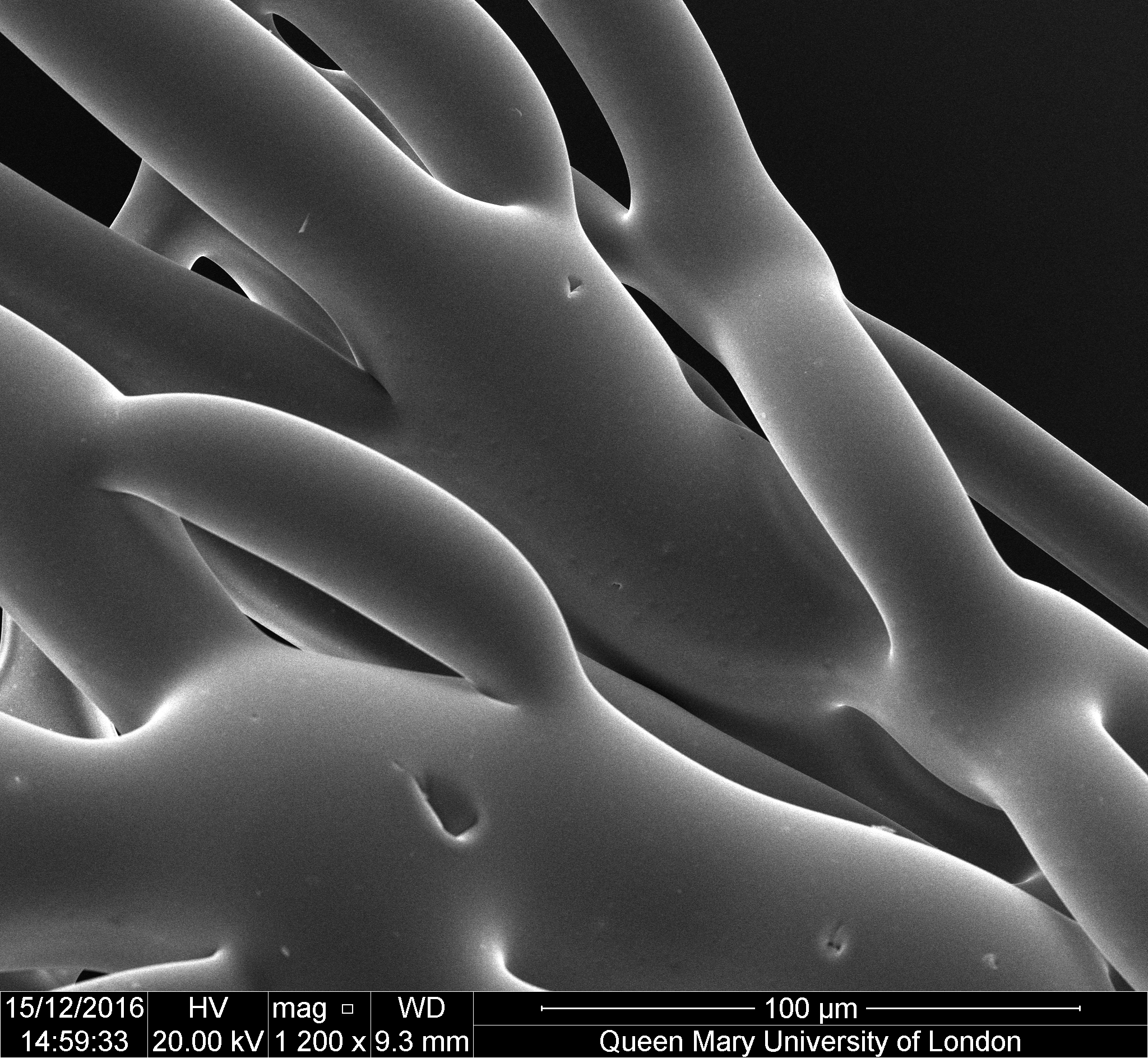
**

a

b

Figure SI2. SEM micrographs of glass wool calcined at 800°C.

**
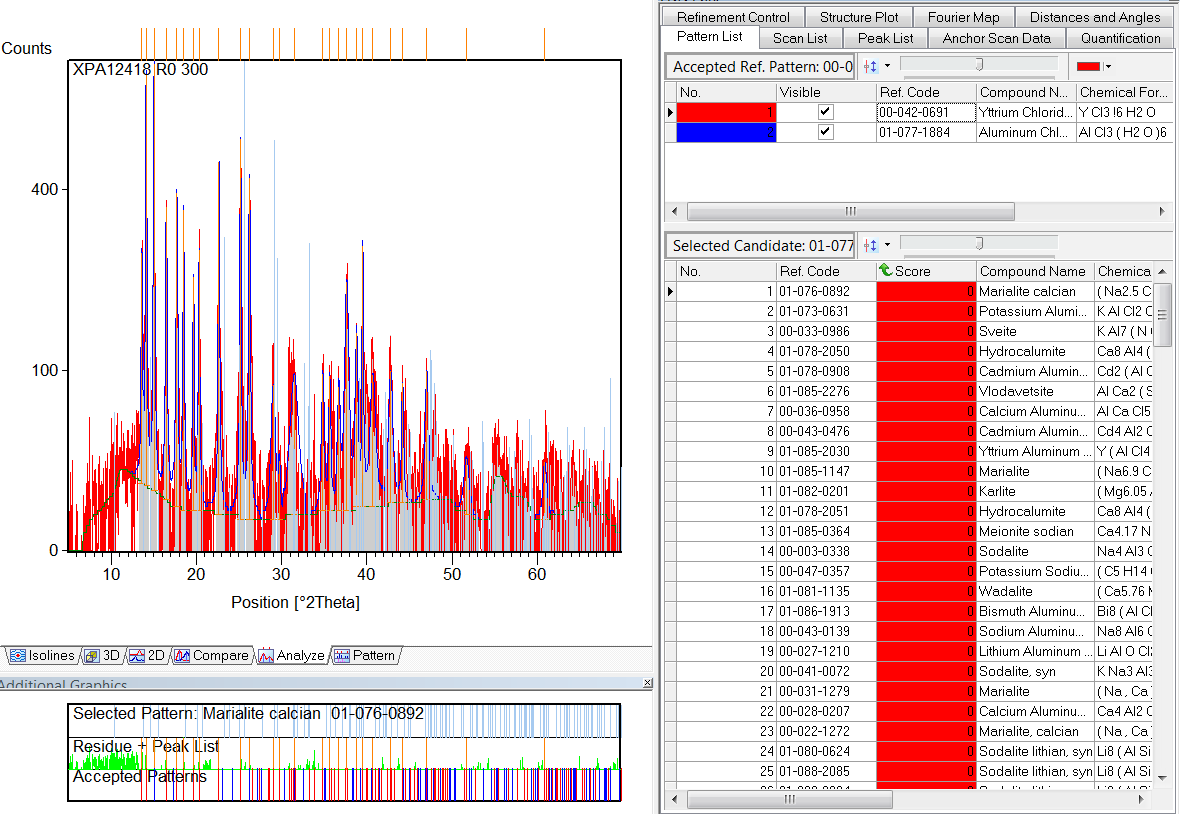
**

Figure SI3. XRD pattern of the sample prepared without addition of urea (R0) and treating at 300°C (R0). YCl_3_·6H_2_O and AlCl_3_ has been identified.

**
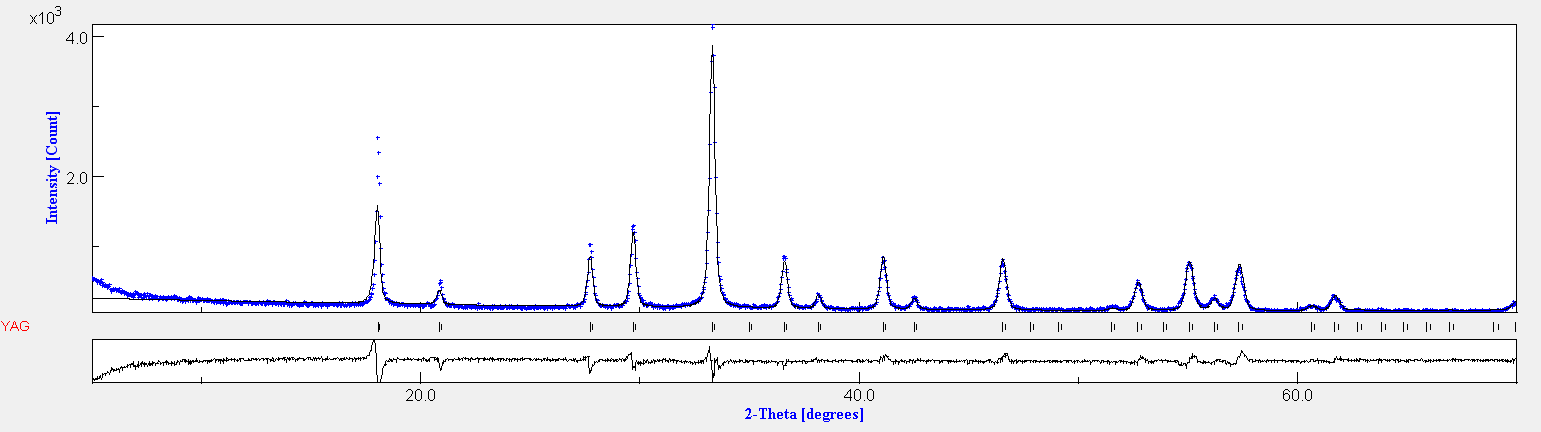
**

Figure SI4. XRD pattern (dots) and the Rietveld fits (full lines) of the sample R1 treated at 900°C. Bar sequences of YAG reference diffraction pattern and residual plot are shown along the bottom.

**
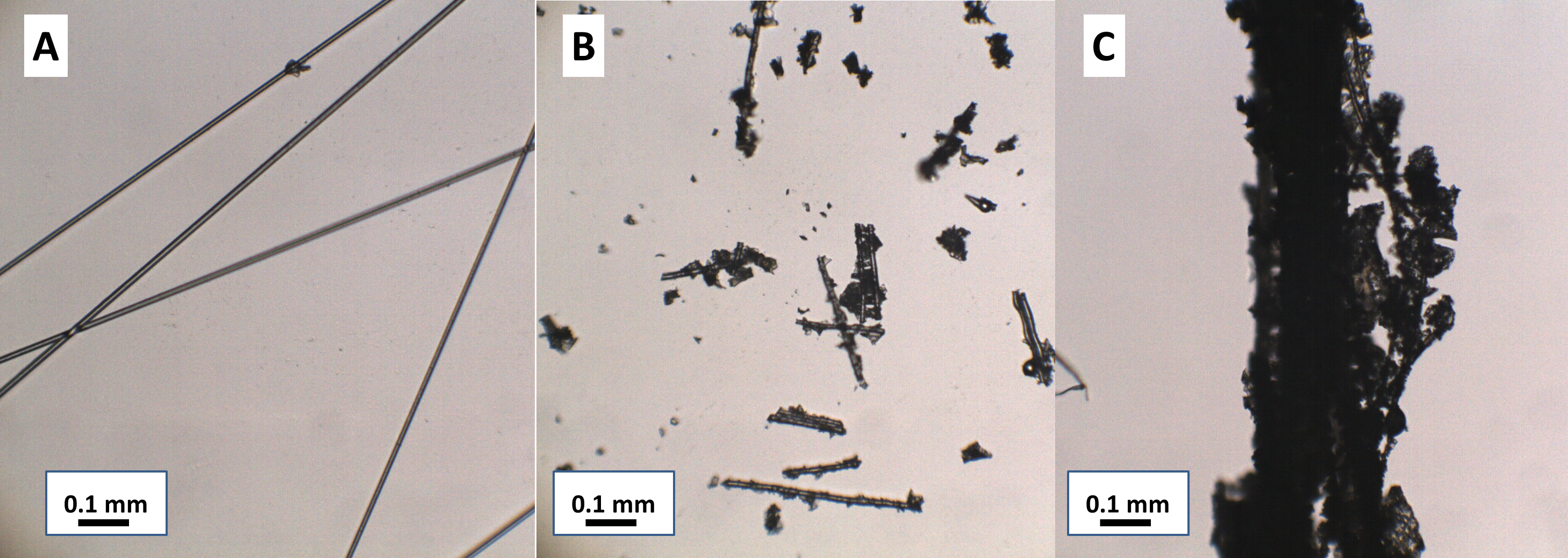
**

Figure SI5. Optical microscope images of (A) glass wool and glass wool template samples obtained at (B) 800°C and (C) 900°C.

**





**

Figure SI6. Particle size distribution obtained from TEM micrographs of glass wool template samples obtained at (A) 800°C and (B) 900°C and (C) of powder obtained at 900°C.

Table I. The quantitative phase determination, cell parameters, crystallite size and lattice microstrain for each phase obtained by applying the Rietveld refinement to the XRD patterns of samples.

| **Starting System** | **Temperature (°C)** | **Phase*** | **%** | **Cell parameters (Å)** | **Crystallite size D (nm)** | **lattice microstrain** |
| --- | --- | --- | --- | --- | --- | --- |
| **R0** | 800 | amorphous | 55.0 (1) | --- | --- | --- |
|  |  | YAG | 44.1 (1) | a = 12.060 (3) | 29 (1) | 0.0033 (1) |
|  | **900** | **YAG** | **91.1 (1)** | **a = 12.052 (4)** | **32 (3)** | **0.00233 (2)** |
|  |  | **YAH** | **8.9 (3)** | **a = 3.674 (9)**  **b = 10.522 (4)** | **32 (1)** | **0.0009 (7)** |
| **R1** | 800 | YAG | 1.90 (1) | a = 12.015 (7) | 45 (8) | err |
|  |  | amorphous | 97.0 (7) | --- | --- | --- |
|  |  | Yttria | 1.10 (8) | a = 10.606 (5) | 41 (9) | 0.0049 (6) |
|  | **900** | **YAG** | **100** | **a = 12.037 (5)** | **65 (2)** | **0.00160 (3)** |
| **R2** | 800 | amorphous | 95.2 (15) | --- | --- | --- |
|  |  | Yttria | 4.7 (3) | a = 10.600 (3) | 26 (3) | 0.0055 (5) |
|  | **900** | **amorphous** | **92.2 (10)** | **---** | **---** | **---** |
|  |  | **Yttria** | **7.8 (2)** | **a = 10.600 (3)** | **25 (1)** | **0.0060 (3)** |

**ICDD phase codes used: YAG 01-079-1891, Yttria 00-025-1200, YAH 01-074-1334*

*# The error is given in parenthesis.*

Table II. The quantitative phase determination, cell parameters, crystallite size and lattice microstrain for each phase obtained by applying the Rietveld refinement to the XRD patterns of the YAG-based nanostructures.

| **Support** | **Temperature (°C)** | **Phase*** | **%** | **Cell parameters (Å)** | **Crystallite size D (nm)** | **lattice microstrain** |
| --- | --- | --- | --- | --- | --- | --- |
| **Powder** | 800 | YAG | 5.7 (3) | a = 12.023 (7) | 46 (2) | err |
|  |  | Yttria | 1.6 (3) | a = 10.620 (1) | 27 (4) | err |
|  |  | amorphous | 92 (12) | ---- | ---- | ---- |
|  | **900** | **YAG** | **100** | **a = 12.037 (5)** | **65 (2)** | **0.00160 (3)** |
| **Paper** | 800 | YAG | 21.4 (2) | a = 12.040 (4) | 46 (4) | 0.0021 (3) |
|  |  | Yttria | 1.4 (4) | a = 10.640 (3) | 27 (9) | err |
|  |  | amorphous | 77 (10) | ---- | ---- | ---- |
|  | 900 | YAG | 60.9(2) | a = 12.111 (7) | 28 (3) | 0.0054 (4) |
|  |  | Yttria | 1.5(1) | a = 10.659 (4) | 35 (9) | 0.002 (1) |
|  |  | YAH | 8.5(3) | a = 3.677 (3)  c = 10.51(1) | 76 (4) | 0.0032 (8) |
|  |  | YAM | 29(10) | a = 7.409 (8)  b = 10.46(1)  c = 11.13(2) | 22 (2) | err |
|  |  | amorphous | 2(1) | ---- | ---- | ---- |
| **Cotton** | 800 | YAG | 82.4 (1) | a = 12.035 (9) | 49 (1) | 0.0017 (1) |
|  |  | Yttria | 7.5 (2) | a = 10.609 (3) | 28 (2) | err |
|  |  | amorphous | 10.1 (5) | ---- | ---- | ---- |
|  | 900 | YAG | 96.53 (1) | a = 12.036 (7) | 42 (2) | 0.00214 (2) |
|  |  | Yttria | 3.47 (9) | a = 10.606(2) | 50 (4) | 0.0017 (2) |
| **Glass wool** | **800** | **YAG** | **100** | **a = 12.030 (8)** | **103 (4)** | **0.00153(7)** |
|  | **900** | **YAG** | **100** | **a = 12.041 (6)** | **74 (1)** | **0.00213(3)** |

**ICDD phase codes used: YAG 01-079-1891, Yttria 00-025-1200, YAH 01-074-1334*

*# The error is given in parenthesis.*

Table III. IR frequencies (cm-1) for the metal-urea starting precursors reported in Figure 10. The wavenumber of pure urea and corresponding attribution are reported for comparison.

| **Assignment** | **Urea** | **R1** | **R2** | **R1 [Y]** | **R1 [Al]** |
| --- | --- | --- | --- | --- | --- |
| δ_s_(NH_2_) | 1678 _m_ | 1650 _st_ | 1648_st_ | 1649_st_ | 1649_st_ |
| δ_as_(NH_2_) | 1616 _sh_ | -- | -- | -- | -- |
| ν(CO) | 1588 _st_ | 1595 _m_ | 1592_m_ | 1592_m_ | 1593_m_ |
| **Legend**: s, as, ν, δ, indicate symmetric, asymmetric, stretching respectively. st, m, sh stand for strong, medium and shoulder. | | | | | |
